# Supplementary material for: Vertical bearing performance of inclined high-pressure rotary spray pile
Source: PLoS One. 2025 Mar 27;20(3):e0319849. doi: 10.1371/journal.pone.0319849 (PMC11949337; doi:10.1371/journal.pone.0319849)
Supplement: S1 Data — (DOCX) [file pone.0319849.s001.docx]

**Minimal Data Set**

**Table 1 Test program**

| Grouping | Number | Angles*/*(°) | *L*/mm | *D*/mm | *L/D* ratio |
| --- | --- | --- | --- | --- | --- |
| T1 | T11 | 0 | 660 | 40 | 16.5 |
|  | T12 | 10 | 660 | 40 | 16.5 |
|  | T13 | 20 | 660 | 40 | 16.5 |
| T2 | T21 | 0 | 860 | 40 | 21.5 |
|  | T22 | 10 | 860 | 40 | 21.5 |
|  | T23 | 20 | 860 | 40 | 21.5 |
| T3 | T31 | 0 | 1060 | 40 | 26.5 |
|  | T32 | 10 | 1060 | 40 | 26.5 |
|  | T33 | 20 | 1060 | 40 | 26.5 |

**Table 2 Indicators of basic physical and mechanical properties of test soil samples**

| $\boldsymbol{\rho}$/(g/cm^3^) | $\boldsymbol{\rho}_{\boldsymbol{dmax}}$/cm^3^) | ***w***/% | ***W_L_***/% | ***W_P_***/% | Cu | Cc |
| --- | --- | --- | --- | --- | --- | --- |
| 1.82 | 1.69 | 35.2 | 59.47 | 25.72 | 6.09 | 0.4 |

Table 3 Settlement change rate of the inclined pile with different loading conditions

| Grouping | Number | Loading  /kN | Settlement/mm | Rate of change  of settlement | L/D ratio sedimentation  rate of change |
| --- | --- | --- | --- | --- | --- |
| T1 | T11 | 4 | 6.062 | / | / |
|  | T12 | 4 | 6.722 | 10.89% | / |
|  | T13 | 4 | 7.232 | 19.30% | / |
| T2 | T21 | 4 | 5.091 | / | 19.07% |
|  | T22 | 4 | 5.722 | 12.39% | 17.47% |
|  | T23 | 4 | 6.086 | 19.54% | 18.83% |
| T3 | T31 | 4 | 4.79 | / | 26.56% |
|  | T32 | 4 | 5.13 | 7.10% | 31.03% |
|  | T33 | 4 | 5.486 | 7.76% | 31.83% |

**Table 4 Grain size distribution of red clay**

| Particle size/mm | Percentage of soil mass smaller than a certain particle size/% |
| --- | --- |
| 10 | 100 |
| 4.75 | 100 |
| 2.0 | 100 |
| 0.425 | 95.06114 |
| 0.25 | 90.58515 |
| 0.075 | 82.41131 |
| 0.02 | 70.8079 |
| 0.002 | 40.131 |

**Fig.4 Grain size distribution of red clay**

**Table 5 Load-displacement relationship curve**

| Loading/kN | Settlement/mm |
| --- | --- |
| 0 | 0 |
| 1 | 1.7005 |
| 2 | 2.4935 |
| 3 | 3.4315 |
| 4 | 4.1865 |
| 5 | 5.436 |
| 6 | 7.385 |
| 7 | 8.1705 |
| 8 | 9.406 |
| 9 | 13.504 |

**Fig. 5 Load-displacement relationship curve**

**Table 6 Change curve of horizontal displacement of pile top**

| Settlement/mm  Loading/kN | T22 | T23 | T11 | T12 | T13 | T31 | T32 | T33 | T22 |
| --- | --- | --- | --- | --- | --- | --- | --- | --- | --- |
| 0 | 0 | 0 | 0 | 0 | 0 | 0 | 0 | 0 | 0 |
| 0.5 | 0 | 0.76 | 1.083 | 0 | 1.37 | 1.603 | 0 | 0.76 | 1.083 |
| 1 | 0 | 0.85 | 1.38 | 0 | 3.8 | 2.9 | 0 | 0.85 | 1.38 |
| 1.5 | 0 | 2.561 | 2.702 | 0 | 4.2 | 3.8 | 0 | 2.561 | 2.702 |
| 2 | 0 | 2.861 | 3.98 | 0 | 5.881 | 5.5 | 0 | 2.861 | 3.98 |
| 2.5 | 0 | 3.903 | 5.327 | 0 | 6.045 | 6.847 | 0 | 3.903 | 5.327 |
| 3 | 0 | 4.609 | 5.639 | 0 | 7.061 | 7.159 | 0 | 4.609 | 5.639 |
| 3.5 | 0 | 6.254 | 6.452 | 0 | 7.894 | 8.372 | 0 | 6.254 | 6.452 |
| 4 | 0 | 6.725 | 6.802 | 0 | 8 | 8.45 | 0 | 6.725 | 6.802 |

**Fig. 6 Change curve of horizontal displacement of pile top**

**Table 7 Variation curve of pile top settlement displacement**

**（a）T11~T13（L/D=16.5）**

| Settlement/mm  Loading/kN | T13 | T12 | T11 |
| --- | --- | --- | --- |
| 0 | 0 | 0 | 0 |
| 0.5 | 0.681 | 0.607 | 0.526 |
| 1 | 0.901 | 0.827 | 0.763 |
| 1.5 | 1.7023 | 1.484 | 1.265 |
| 2 | 3.043 | 2.897 | 2.238 |
| 2.5 | 4.282 | 3.487 | 3.306 |
| 3 | 4.921 | 4.139 | 3.628 |
| 3.5 | 5.868 | 5.422 | 5.091 |
| 4 | 7.232 | 6.722 | 6.062 |

**（b）T21~T23（L/D=21.5）**

| Settlement/mm  Loading/kN | T21 | T22 | T23 |
| --- | --- | --- | --- |
| 0 | 0 | 0 | 0 |
| 0.5 | 0.436 | 0.487 | 0.581 |
| 1 | 0.763 | 0.827 | 0.901 |
| 1.5 | 0.998 | 1.047 | 1.141 |
| 2 | 1.265 | 1.484 | 1.6023 |
| 2.5 | 1.703 | 1.997 | 2.411 |
| 3 | 2.628 | 3.139 | 3.921 |
| 3.5 | 3.261 | 3.872 | 4.868 |
| 4 | 5.091 | 5.722 | 6.086 |

**（c）T31~T33（L/D=26.5）**

| Settlement/mm  Loading/kN | T31 | T32 | T33 |
| --- | --- | --- | --- |
| 0 | 0 | 0 | 0 |
| 0.5 | 0.663 | 0.738 | 0.809 |
| 1 | 1.056 | 1.304 | 1.547 |
| 1.5 | 1.826 | 2.348 | 2.73 |
| 2 | 2.036 | 2.557 | 3.082 |
| 2.5 | 2.497 | 3.057 | 3.411 |
| 3 | 3.439 | 3.628 | 4.142 |
| 3.5 | 4.019 | 4.209 | 4.522 |
| 4 | 4.79 | 5.13 | 5.486 |

**（a）T11~T13（L/D=16.5）**

**（b）T21~T23（L/D=21.5）**

**（c）T31~T33（L/D=26.5）**

**Fig. 7 Variation curve of pile top settlement displacement**

**Table 8 Variation curve of pile axial force along depth**

**（a）L/D=16.5**

| kN  depth | T11 | T12 | T13 | T11 | T12 | T13 | T11 | T12 | T13 | T11 | T12 | T13 |
| --- | --- | --- | --- | --- | --- | --- | --- | --- | --- | --- | --- | --- |
| 0 | 1 | 1 | 1 | 2 | 2 | 2 | 3 | 3 | 3 | 4 | 4 | 4 |
| 0.14 | 0.72 | 0.62 | 0.603 | 1.572 | 1.429 | 1.428 | 2.86 | 2.515 | 2.272 | 3.38 | 3.24 | 3.167 |
| 0.28 | 0.602 | 0.528 | 0.521 | 1.345 | 1.22 | 1.19 | 2.235 | 2.041 | 1.922 | 2.939 | 2.74 | 2.682 |
| 0.42 | 0.573 | 0.474 | 0.442 | 0.998 | 0.903 | 0.907 | 1.604 | 1.571 | 1.469 | 2.498 | 2.192 | 2.14 |
| 0.56 | 0.472 | 0.421 | 0.367 | 0.508 | 0.542 | 0.528 | 1.252 | 1.028 | 1.124 | 1.925 | 1.608 | 1.572 |
| 0.7 | 0.29 | 0.266 | 0.207 | 0.405 | 0.304 | 0.43 | 0.907 | 0.823 | 0.804 | 1.415 | 1.231 | 1.145 |
| 0.85 | 0.11 | 0.12 | 0.092 | 0.21 | 0.181 | 0.174 | 0.625 | 0.502 | 0.43 | 0.829 | 0.809 | 0.8 |

**（b）L/D=21.5**

| kN  depth | T21 | T22 | T23 | T21 | T22 | T23 | T21 | T22 | T23 | T21 | T22 | T23 |
| --- | --- | --- | --- | --- | --- | --- | --- | --- | --- | --- | --- | --- |
| 0 | 1 | 1 | 1 | 2 | 2 | 2 | 3 | 3 | 3 | 4 | 4 | 4 |
| 0.14 | 0.812 | 0.82 | 0.803 | 1.272 | 1.238 | 1.528 | 2.786 | 2.415 | 2.272 | 3.258 | 3.15 | 3.074 |
| 0.28 | 0.621 | 0.528 | 0.521 | 1.044 | 1.02 | 1.09 | 2.035 | 2.041 | 1.982 | 2.739 | 2.44 | 2.382 |
| 0.42 | 0.473 | 0.474 | 0.442 | 0.998 | 0.903 | 0.977 | 1.804 | 1.71 | 1.169 | 2.398 | 2.092 | 2.195 |
| 0.56 | 0.372 | 0.121 | 0.286 | 0.508 | 0.542 | 0.528 | 1.32 | 1.228 | 1.174 | 1.825 | 1.58 | 1.572 |
| 0.7 | 0.19 | 0.066 | 0.107 | 0.205 | 0.204 | 0.3 | 1.07 | 0.823 | 0.804 | 1.605 | 1.231 | 1.145 |
| 0.85 | 0.011 | 0.02 | 0.012 | 0.11 | 0.11 | 0.14 | 0.625 | 0.602 | 0.605 | 1.029 | 0.997 | 0.837 |

**（c）L/D=26.5**

| kN  depth | T31 | T32 | T33 | T31 | T32 | T33 | T31 | T32 | T33 | T31 | T32 | T33 |
| --- | --- | --- | --- | --- | --- | --- | --- | --- | --- | --- | --- | --- |
| 0 | 1 | 1 | 1 | 2 | 2 | 2 | 3 | 3 | 3 | 4 | 4 | 4 |
| 0.14 | 0.622 | 0.592 | 0.565 | 1.659 | 1.56 | 1.41 | 2.323 | 2.248 | 2.069 | 3.238 | 3.185 | 3.268 |
| 0.28 | 0.378 | 0.426 | 0.42 | 0.919 | 1.074 | 0.91 | 1.609 | 1.56 | 1.34 | 2.579 | 2.548 | 2.335 |
| 0.42 | 0.253 | 0.289 | 0.301 | 0.616 | 0.805 | 0.61 | 1.127 | 1.142 | 0.86 | 1.96 | 1.614 | 1.509 |
| 0.56 | 0.105 | 0.17 | 0.17 | 0.408 | 0.304 | 0.271 | 0.806 | 0.712 | 0.51 | 1.6867 | 1.314 | 1.078 |
| 0.7 | 0.033 | 0.069 | 0.081 | 0.176 | 0.242 | 0.128 | 0.403 | 0.643 | 0.476 | 1.2051 | 0.885 | 0.83 |
| 0.85 | 0.015 | 0.021 | 0.033 | 0.064 | 0.074 | 0.08 | 0.363 | 0.351 | 0.343 | 0.73 | 0.61 | 0.63 |

**（a）L/D=16.5**

**（b）L/D=21.5**

**（c）L/D=26.5**

**Fig. 8 Variation curve of pile axial force along depth**

**Table 9 Change curve of pile bending moment**

| N·m  depth | T12 | T13 | T22 | T23 | T32 | T33 |
| --- | --- | --- | --- | --- | --- | --- |
| 0 | 0 | 0 | 0 | 0 | 0 | 0 |
| 0.14 | 1.69794 | 1.8727 | 1.81469 | 3.19975 | 2.69944 | 2.98291 |
| 0.28 | 6.73389 | 8.92212 | 7.19975 | 9.892 | 8.15654 | 10.72108 |
| 0.42 | 2.2772 | 3.88822 | 3.49205 | 1.98098 | 3.41603 | 7.59788 |
| 0.56 | 0.57952 | 1.38644 | 0.31556 | 0.49 | 0.86744 | -0.13 |
| 0.7 | 0.05372 | 0.18007 | 0.05074 | 0.32576 | 0.58178 | 0.517 |
| 0.85 | 0.05584 | 0.16007 | 0.01038 | 0.05074 | 0.58 | 0.5176 |

**Fig. 9 Change curve of pile bending moment**

**Table 10 Variation curve of pile shear force with depth**

| N  depth | T12 | T13 | T22 | T23 | T32 | T33 |
| --- | --- | --- | --- | --- | --- | --- |
| 0 | 106.41 | 146.41 | 189.53 | 242.64 | 208.98 | 258.01 |
| 0.14 | -105 | -145 | -105 | -151 | -114 | -173 |
| 0.28 | -94 | -94 | -88 | -90 | -43 | -75 |
| 0.42 | 0 | 0 | 0 | 0 | 0 | 0 |
| 0.56 | 0 | 0 | 0 | 0 | 0 | 0 |
| 0.7 | 0 | 0 | 0 | 0 | 0 | 0 |
| 0.85 | 0 | 0 | 0 | 0 | 0 | 0 |

**Fig. 10 Variation curve of pile shear force with depth**

**Table 11 Variation curve of lateral friction resistance of pile along the depth**

**（a）L/D=16.5**

| kPa  depth | T11 | T12 | T13 | T11 | T12 | T13 | T11 | T12 | T13 | T11 | T12 | T13 |
| --- | --- | --- | --- | --- | --- | --- | --- | --- | --- | --- | --- | --- |
| 0.14 | 9.2929 | 30.25 | 31.608 | 34.076 | 45.461 | 18.5414 | 48.1465 | 38.61465 | 57.96178 | 49.36306 | 60.50955 | 66.32166 |
| 0.28 | 9.3949 | 7.324 | 6.5286 | 18.073 | 16.640 | 18.9490 | 49.76115 | 37.73885 | 27.86624 | 49.36306 | 39.80892 | 38.61465 |
| 0.42 | 2.3089 | 4.299 | 6.2898 | 27.627 | 25.238 | 22.5318 | 50.23885 | 37.42038 | 36.06688 | 49.36306 | 43.63057 | 43.15287 |
| 0.56 | 8.0414 | 4.219 | 5.9713 | 39.012 | 28.742 | 30.1751 | 28.02548 | 43.23248 | 27.46815 | 49.36306 | 46.49682 | 45.22293 |
| 0.7 | 14.490 | 12.34 | 12.738 | 8.2006 | 18.949 | 7.80255 | 27.46815 | 16.32166 | 25.47771 | 49.36306 | 30.01592 | 33.99682 |
| 0.85 | 14.331 | 11.62 | 9.1560 | 15.525 | 9.7929 | 20.3821 | 22.45223 | 25.55732 | 29.77707 | 49.36306 | 33.59873 | 27.46815 |

**（b）L/D=21.5**

| kPa  depth | T21 | T22 | T23 | T21 | T22 | T23 | T21 | T22 | T23 | T21 | T22 | T23 |
| --- | --- | --- | --- | --- | --- | --- | --- | --- | --- | --- | --- | --- |
| 0.14 | 11.513 | 11.024 | 12.0651 | 44.57986 | 46.6505 | 28.907 | 30.106 | 35.8280 | 44.58599 | 45.443 | 52.0578 | 56.7124 |
| 0.28 | 11.697 | 17.883 | 17.2709 | 13.99743 | 13.369 | 26.825 | 45.994 | 22.9054 | 17.760 | 31.7858 | 43.4835 | 42.3811 |
| 0.42 | 9.0641 | 3.3072 | 4.83831 | 2.7896 | 7.1656 | 6.9206 | 14.147 | 20.2719 | 49.7917 | 20.8843 | 21.3130 | 11.4282 |
| 0.56 | 6.1856 | 21.619 | 9.55414 | 30.01 | 22.109 | 27.4987 | 29.642 | 29.5198 | 30 | 35.0930 | 31.3571 | 38.179 |
| 0.7 | 11.1465 | 3.3684 | 10.9627 | 18.557 | 20.7006 | 13.9637 | 15.311 | 24.8040 | 22.6604 | 13.4737 | 21.3743 | 26.151 |
| 0.85 | 10.9627 | 2.8172 | 5.8182 | 5.8182 | 5.75698 | 9.7991 | 27.253 | 13.5350 | 12.1876 | 35.2768 | 14.3312 | 18.863 |

**（c）L/D=26.5**

| kPa  depth | T31 | T32 | T33 | T31 | T32 | T33 | T31 | T32 | T33 | T31 | T32 | T33 |
| --- | --- | --- | --- | --- | --- | --- | --- | --- | --- | --- | --- | --- |
| 0.14 | 18.8047 | 20.3025 | 21.646 | 16.9536 | 21.8949 | 29.35908 | 33.6883 | 37.4203 | 46.32763 | 37.9179 | 40.5553 | 36.42516 |
| 0.28 | 12.1467 | 8.2603 | 7.2153 | 36.7943 | 24.1839 | 24.88057 | 35.5294 | 34.2356 | 36.27588 | 32.7926 | 31.6978 | 46.42715 |
| 0.42 | 6.22014 | 6.8172 | 5.9215 | 15.0952 | 13.3857 | 14.92834 | 23.9848 | 20.8001 | 23.88535 | 30.8021 | 46.4769 | 41.10271 |
| 0.56 | 7.36465 | 5.9215 | 6.5187 | 10.3473 | 24.9303 | 16.86903 | 15.9733 | 21.3972 | 17.4164 | 13.5867 | 14.9283 | 21.44705 |
| 0.7 | 3.5828 | 5.0258 | 4.4287 | 11.5669 | 3.08519 | 7.11584 | 20.0537 | 3.43352 | 1.69188 | 23.9779 | 21.3475 | 12.34076 |
| 0.85 | 0.8957 | 2.3885 | 2.3885 | 5.55583 | 8.35987 | 2.38854 | 1.99045 | 14.5302 | 6.61823 | 23.6415 | 13.6843 | 9.95223 |

**（a）L/D=16.5**

**（b）L/D=21.5**

**（c）L/D=26.5**

**Fig. 11 Variation curve of lateral friction resistance**
