# Supplementary material for: Vertical bearing performance of inclined high-pressure rotary spray pile
Source: PLoS One. 2025 Mar 27;20(3):e0319849. doi: 10.1371/journal.pone.0319849 (PMC11949337; doi:10.1371/journal.pone.0319849)
Supplement: Attachment — Submitted filename: Fig. 4.pdf [file pone.0319849.s004.pdf]

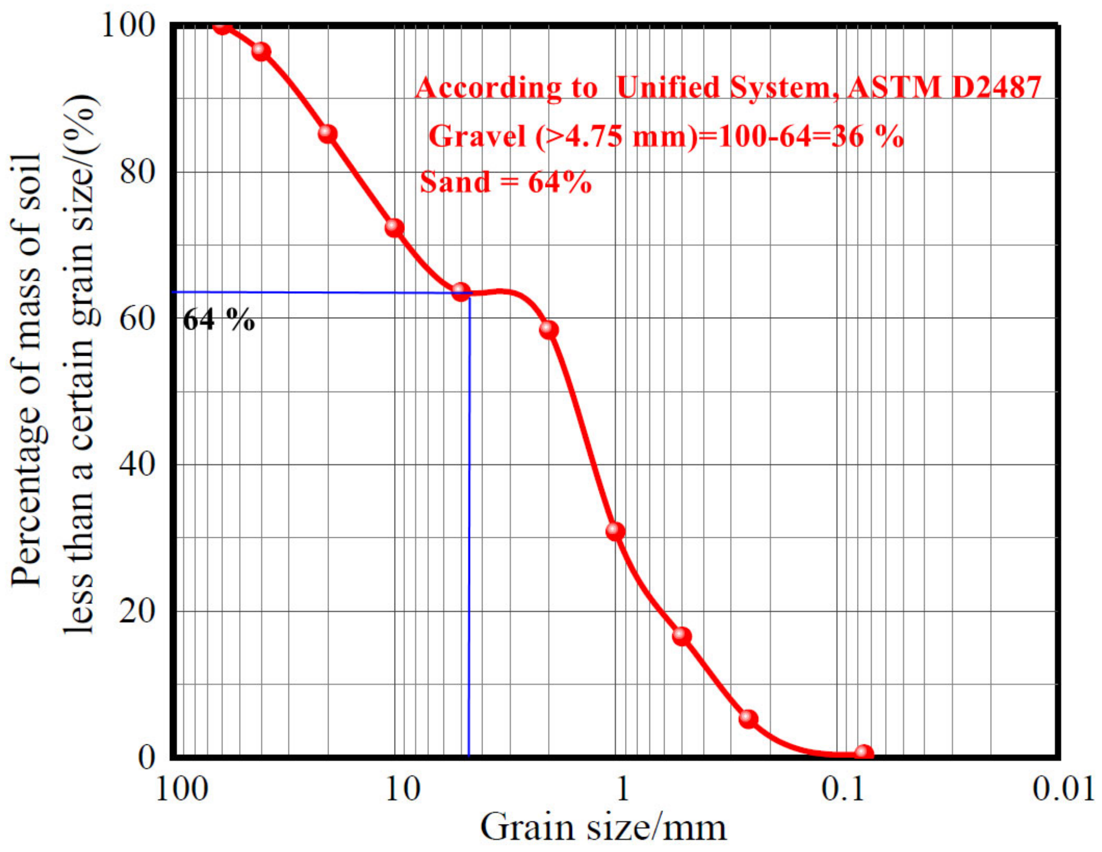


Fig. 4

Dear Author(s)
In figure 4, the soil is gravelly sand, not clay soil, according to Unified Soil Classification System (ASTM D2487-17e1)
Gravel (>4.75 mm) = 100-64 = 36%
Sand = 64%
Clay = 0 %
Please redraw the figure so that the passage through the No. 200 sieve is more than 50% according to the unified classification system, or set the grain sizes of gravel, sand and clay according to Chinese specifications
