# Supplementary material for: Vertical bearing performance of inclined high-pressure rotary spray pile
Source: PLoS One. 2025 Mar 27;20(3):e0319849. doi: 10.1371/journal.pone.0319849 (PMC11949337; doi:10.1371/journal.pone.0319849)
Supplement: Attachment — Submitted filename: Response to Reviewers.docx [file pone.0319849.s002.docx]

Dear editor:
On behalf of my co-authors, we thank you very much for giving us an opportunity to revise our manuscript, we appreciate editor and reviewers very much for their positive and constructive comments and suggestions on our manuscript entitled “Research on vertical bearing performance of inclined high-pressure rotary spray pile”. (ID: PONE-D-24-43810).
We have studied reviewer’s comments carefully and have made revisions which are marked in red in the paper. We have tried our best to revise our manuscript according to the comments and journal formatting guidelines. Attached please find the revised version, which we would like to submit for your kind consideration.
We would like to express our great appreciation to you and reviewers for comments on our paper. We are looking forward to hearing from you.
Thank you and best regards.
Yours sincerely,
Fei Gan
Corresponding author:
Name: Fei Gan
E-mail: [fgan@gzu.edu.cn](mailto:fgan@gzu.edu.cn)

List of Responses
Dear Editors and Reviewers:
Thank you for your letter and for the reviewers’ comments concerning our manuscript entitled “Research on vertical bearing performance of inclined high-pressure rotary spray pile”. (ID: PONE-D-24-43810). Those comments are all valuable and very helpful for revising and improving our paper, as well as the important guiding significance to our research. We have studied comments carefully and have made corrections which we hope meet with approval. Revised portions[1] are marked in red or blue in the paper. Original text in red italics. The main corrections in the paper and the responses to the reviewer’s comments are as follows:

Response to Academic Editorial Editor:

1. When submitting your revision, we need you to address these additional requirements. Please ensure that your manuscript meets PLOS ONE's style requirements, including those for file naming.

Response: We thank the editors for their comments. We have reorganized the paper according to the PLOS One style.

2. We note that the grant information you provided in the ‘Funding Information’ and ‘Financial Disclosure’ sections do not match.

When you resubmit, please ensure that you provide the correct grant numbers for the awards you received for your study in the ‘Funding Information’ section.

Response: Thank you very much for your reminder. We have corrected the correct funding number.

3. We note that your Data Availability Statement is currently as follows: All relevant data are within the manuscript and its Supporting Information files.

Please confirm at this time whether or not your submission contains all raw data required to replicate the results of your study. Authors must share the “minimal data set” for their submission. PLOS defines the minimal data set to consist of the data required to replicate all study findings reported in the article, as well as related metadata and methods (<https://journals.plos.org/plosone/s/data-availability#loc-minimal-data-set-definition>).

Response: Thank you very much for your reminder. We have uploaded the 'minimal data set'.

**Responds to the reviewer’s comments:
Reviewer #1:**

1. Poor English

Response: Thank you very much for your suggestion. We tried our best to improve the manuscript and made some changes to the manuscript. These changes will not influence the content and framework of the paper. Here we did not list the changes but marked in red in the revised paper, We appreciate for Reviewers' work earnestly and hope that the correction will meet with approval.

2. Long Abstract, unreflective and general keywords

Response: Thank you very much for suggesting that I pay attention to and refine this by revising the abstracts to be concise enough that some of the experimental results can be seen in the abstracts. I have revised the keywords to accurately reflect the content of the paper.

*Abstract: Inclined piles are gradually being used in large-scale transmission line tower foundation projects, To study the bearing deformation properties and load transfer mechanism of inclined piles under vertical loading, nine single inclined pile tests were carried out in red clay soil to analyze the effects of pile inclination and length-to-diameter ratio on the axial force, bending moment, shear force, and lateral friction force of inclined piles. The test results show that：(1) Under the action of vertical load at the top of the pile, the larger the inclination angle, the larger the settlement of the inclined pile; (2) The axial force of the inclined pile is smaller than that of the corresponding vertical pile;(3) Bending moment and shear force exist in the upper section of the inclined pile, and the maximum bending moment of the inclined pile is affected by the inclination angle of the pile body and the length-to-diameter ratio;(4) In the upper section of the pile, the average pile-side molecular resistance of inclined piles is greater than that of vertical piles, and the maximum molecular resistance of inclined piles all occurs in the section from the top of the pile to the pile with the relative depth Z/L=0.14.*

*Keywords: Pile foundation; Inclined pile; Bearing deformation properties; Model test; Pile inclination; L/D ratio*

（Please see page 1, lines 8-21.）

3. Superficial technical review: Uncharacterized research gaps/not critically analyzed relevant updated works/not outlined the main innovation level in the science border in comparison with other studies.

Response: Thank you very much for your careful reading. We have rewritten the introduction in the revised manuscript based on your feedback. We sincerely appreciate the enthusiastic work of the reviewers and hope to receive your approval for the corrections.

（Please see pages 1-3, lines 23-61.）

4. Which type of foundation???

Response: Thank you very much for the reminder, I have highlighted my research in the abstract as well as in the keywords; pile foundation.

（See lines 8-21on page 1 in the article）

5. Doesn’t have any convincing and documented Discussion in terms of evidential analysis/solid comparison with other scholars' approaches/uncertainty quantification/technical limitations/pitfalls and practical difficulties/the impact of the used data and involved noises on the results/ …

Response: Thank you very much for your valuable suggestions, which were carefully considered, and the authors thank the reviewers for their comments, comparing their research results with others to obtain similar conclusions. Relevant literature was also cited.

*[40].	Zhao M, Cao WP, Li H (2023) Model test of vertical compressive bearing deformation properties of inclined piles. Municipal technology. 41(11) 94-100. https://doi.org/10.19922/j.1009-7767.2023.11.094.*

*[41].	Cao WP, Qin QY, Zhao M (2019) Model tests on uplift behaviors of single batter piles in sand. Chinese Journal of Rock Mechanics and Engineering. 38(S1) 3124-3133.*

*[42].	Cao WP, Lu QY, Fan WF, Li S (2016) Experimental Study on Load Transfer Characteristics of Sloping Piles under Vertical Loads. Rock and Soil Mechanics. 37(11) 3048-3056.* [*https://doi.org/10.16285/j.rsm.2016.11.002*](https://doi.org/10.16285/j.rsm.2016.11.002)*.*

（See lines 511-517on page 23 in the article）

6. Doesn’t consider the spatial soil type distribution mapping and corresponding effects on the results (for example, https://link.springer.com/article/10.1007/s10064-018-1400-9...)

Response: Thank you very much for the suggestion that reminded us of factors we had not taken into account, so we mentioned in the introduction about the spatial soil type distribution map and the corresponding impact on the results and cited the relevant literature.

*[25].	Ghaderi A, Shahri A A, Larsson S (2019) An artificial neural network based model to predict spatial soil type distribution using piezocone penetration test data (CPTu). BULLETIN OF ENGINEERING GEOLOGY AND THE ENVIRONMENT. 78(6) 4579-4588. https://doi.org/10.1007/s10064-018-1400-9.*

（See lines 469 to 471 on page 21 of the article）

7. The effect of ground water is neglected.

Response: Thank you very much for your suggestion. It mentioned factors that were not considered in our article, and we will focus on the impact of groundwater in future research. And it has been pointed out in the article.

*It should be noted that in this paper, compressive tests were conducted only on inclined piles under free conditions at the top of the pile in red clay with certain water content and heaviness, and the effect of groundwater was not considered. More tests are needed to investigate the compressive properties of inclined piles in other states of cohesive soils as well as in cohesionless soils with different displacement boundary conditions at the top of the pile.*

（See article, pages 18-19, lines 388-392）

8. The effect of clay sensitivity on engineering results is not evaluated (for example https://www.sciencedirect.com/science/article/abs/pii/S0013795215000411, https://link.springer.com/article/10.1007/s10706-016-9976-y …)

Response: Thank you very much for your suggestion to draw our attention to factors that we have not considered, so we have mentioned the effect of clay sensitivity on engineering results in the introduction and cited the relevant literature.

*[26].	Shahri A A, Malehmir A, Juhlin C (2015) Soil classification analysis based on piezocone penetration test data - A case study from a quick-clay landslide site in southwestern Sweden. ENGINEERING GEOLOGY. 199 167. https://doi.org/10.1016/j.enggeo.2015.01.022.*

*[27].	Shahri A A (2016) An Optimized Artificial Neural Network Structure to Predict Clay Sensitivity in a High Landslide Prone Area Using Piezocone Penetration Test (CPTu) Data: A Case Study in Southwest of Sweden. Geotechnical and Geological Engineering. https://doi.org/10.1007/s10706-016-9976-y.*

*[28].	Shahri A A (2016) Assessment and Prediction of Liquefaction Potential Using Different Artificial Neural Network Models: A Case Study. Geotechnical and Geological Engineering. 34 807–815. https://doi.org/10.1007/s10706-016-0004-z.*

*[29].	Shahri A A, Malehmir A, Juhlin C (2015) Malehmir and C. Juhlin, Soil classification analysis based on piezocone penetration test data - A case study from a quick-clay landslide site in southwestern Sweden. ENGINEERING GEOLOGY. 189 32-47. https://doi.org/10.1016/j.enggeo.2015.01.022.*

（See pages 21-22 of the article, lines 472~483）

9. Ill-formatted reference list in terms of format, identifiers, DOI…

Response: We apologize for our carelessness. The incorrectly formatted reference list was corrected in our resubmitted manuscript. Thank you for the correction.

（See pages 19-23 of the article, lines 403~517）

10. Unjustified conclusion

Response: Thank you for the reviewer's comments. We have rewritten the conclusion to include important numerical findings from the study, making it more justified.

*(1) When the vertical load at the top of the pile and the length-to-diameter ratio are certain, the vertical bearing performance of vertical piles is the best; the resulting settlement is less than that of inclined piles of 10° and 20°. When the vertical load and angle at the top of the pile are certain and L/D=26.5, the inclined pile has the best vertical bearing performance and produces less settlement than the inclined pile with L/D=16.5 and L/D=21.5.*

*(2) The bending moment of the pile under different working conditions reaches the maximum value when Z/L=0.24, and the larger the inclination angle of the pile body at the shallower position with the same length-diameter ratio, the more obvious the change rule of the bending moment of the pile. The pile bending moment gradually tends to zero at deeper locations.*

*(3) The maximum shear of an inclined pile is in the top section of the pile, and extreme shear exists in the pile at a certain depth below the top. For this paper, this extreme shear is approximately at Z/L = 0.14. The greater the L/D ratio and pile inclination, the greater the pile shear at the same depth.*

*(4) Regardless of the vertical load, pile inclination, and L/D ratio, the maximum frictional resistance of inclined piles occurs in the section from the top of the pile to the pile with a relative depth of Z/L = 0.14. In the upper section of the pile, the average pile-side friction resistance of the inclined pile is greater than that of the vertical pile. In contrast, in the lower section of the pile, the average lateral friction resistance on the side of the inclined pile is not much different from that of the vertical pile in general.*

（See page 18 of the article, lines 371~387）

Reviewer # 2:

1- Rewrite the abstract so that it is limited to presenting the problem of the research and the results reached by the researcher.

Response: Thank you very much for your valuable suggestions, we have rewritten the abstract based on the reviewers' suggestions.

*Abstract: Inclined piles are gradually being used in large-scale transmission line tower foundation projects, To study the bearing deformation properties and load transfer mechanism of inclined piles under vertical loading, nine single inclined pile tests were carried out in red clay soil to analyze the effects of pile inclination and length-to-diameter ratio on the axial force, bending moment, shear force, and lateral friction force of inclined piles. The test results show that：(1) Under the action of vertical load at the top of the pile, the larger the inclination angle, the larger the settlement of the inclined pile; (2) The axial force of the inclined pile is smaller than that of the corresponding vertical pile;(3) Bending moment and shear force exist in the upper section of the inclined pile, and the maximum bending moment of the inclined pile is affected by the inclination angle of the pile body and the length-to-diameter ratio;(4) In the upper section of the pile, the average pile-side molecular resistance of inclined piles is greater than that of vertical piles, and the maximum molecular resistance of inclined piles all occurs in the section from the top of the pile to the pile with the relative depth Z/L=0.14.*

（See lines 8-19 on page 1）

2;2.1a. What are the criteria for selecting dimensions of soil tank?

Response: Thank you very much for your question. We conducted a single high-pressure rotary jet grouting pile compression field test at the Chunfengjing project in Gui'an New District, Guiyang City. The size of the soil tank was reduced proportionally based on the field test, approximately equal to 1.2m × 1.2m × 1.5m.

3;2.1b. Why didn't the researcher use natural soil in the physics model?

Response: Thank you very much for the reviewer's question. The red clay used in this experiment is taken from a natural slope and has similar soil parameters and mechanical properties to those tested on site. Therefore, the experimental data obtained is more in line with actual laws.

4; 2.3a. The model piles were made by cast-in-place slurry reinforcement in hydraulic soil?

Response: Thank you very much for your question, the model piles are made by pouring the soil on-site, in order to ensure the strength also needs to be maintained for 7 days so that the model piles can also be made to increase the friction factor of the surface.

5 Table 2 Standard for each test???

Response: Thank you very much for your question, have added the relevant content in the text; Table 2 test of the standard is based on the “geotechnical test method standard” (GB/T 50123-2019) to measure the basic physical indicators of soil samples. And the relevant norms are cited.

*[35].	China Ministry Of Water Resources, Geotechnical test method standards. 2019, China Planning Press: Beijing.*

（See lines 499-500 on page 22）

6. 3.2. Fig.4 Where is the clay? Note: the soil sample is sand according to grain size distribution curve

Response: I am very grateful to the reviewer for the reminder and have changed the grain size grading curve for sandy soils to that of red clays

（See line 119 on page 6 of the article）

7. 3.2 3.2 Vertical bearing capacity to 3.2 bearing capacity

Response: Thank you very much for your question. The text does not have a direct value for the size of the bearing capacity but rather compares the size of the settlement under the same load to determine the bearing performance of inclined piles at different angles. Under the same size of vertical load, the one with the smaller settlement is considered to have better bearing performance.

*For the same length-to-diameter ratio, the larger the inclination angle of the pile body, the worse the vertical bearing performance of the pile foundation. For example, when L/D=16.5, under the steady state of pile top settlement, T11, T12, and T13 groups are 6.062, 6.722, and 7.232 mm, respectively, and compared with the vertical pile T11 group, the vertical settlements of the T12 and T13 groups are increased by 10.89% and 19.30%, respectively, compared with that of the T11 group; It can be seen that under the same test conditions, the larger the inclination angle of the inclined pile is, the larger the vertical displacement of the top of the pile is. which indicates that reducing the angle to a certain extent can increase its vertical bearing performance, i.e., the vertical compressive ultimate bearing capacity of inclined piles is smaller than that of vertical piles*

（See page 9 of the article, lines 201 to 209.）

8. Figures 7, change the Y axis title to Settlement

Response: Thank you for your suggestion. The Y-axis title has been changed to 'Settlement'.

（See page 10 of the article, lines 210 to 215.）

9; 3.3 Eq. 1 (Ref.?)

We greatly appreciate the reviewer's reminder and have added relevant literature to explain the source of Formula 1, as well as the sources of Formulas 2, 3, and 4.

*[41].	Cao WP, Qin QY, Zhao M (2019) Model tests on uplift behaviors of single batter piles in sand. Chinese Journal of Rock Mechanics and Engineering. 38(S1) 3124-3133.*

（See page 23 of the article, lines 513 to 514.）

10. 4 Conclusion

Rewrite the conclusions to include the most important numerical findings of the study

Response: Thank you very much, we have rewritten the conclusion based on your valuable comments to include the important numerical findings of the study.

*(1) When the vertical load at the top of the pile and the length-to-diameter ratio are certain, the vertical bearing performance of vertical piles is the best; the resulting settlement is less than that of inclined piles of 10° and 20°. When the vertical load and angle at the top of the pile are certain and L/D=26.5, the inclined pile has the best vertical bearing performance and produces less settlement than the inclined pile with L/D=16.5 and L/D=21.5.*

*(2) The bending moment of the pile under different working conditions reaches the maximum value when Z/L=0.24, and the larger the inclination angle of the pile body at the shallower position with the same length-diameter ratio, the more obvious the change rule of the bending moment of the pile. The pile bending moment gradually tends to zero at deeper locations.*

*(3) The maximum shear of an inclined pile is in the top section of the pile, and extreme shear exists in the pile at a certain depth below the top. For this paper, this extreme shear is approximately at Z/L = 0.14. The greater the L/D ratio and pile inclination, the greater the pile shear at the same depth.*

*(4) Regardless of the vertical load, pile inclination, and L/D ratio, the maximum frictional resistance of inclined piles occurs in the section from the top of the pile to the pile with a relative depth of Z/L = 0.14. In the upper section of the pile, the average pile-side friction resistance of the inclined pile is greater than that of the vertical pile. In contrast, in the lower section of the pile, the average lateral friction resistance on the side of the inclined pile is not much different from that of the vertical pile in general.*

（See page 18 of the article, lines 371 to 387.）
