# Supplementary material for: Vertical bearing performance of inclined high-pressure rotary spray pile
Source: PLoS One. 2025 Mar 27;20(3):e0319849. doi: 10.1371/journal.pone.0319849 (PMC11949337; doi:10.1371/journal.pone.0319849)
Supplement: Attachment — Submitted filename: Response_to_Reviewers_auresp_2.docx [file pone.0319849.s003.docx]

Dear editor:
On behalf of my co-authors, we thank you very much for giving us an opportunity to revise our manuscript, we appreciate editor and reviewers very much for their positive and constructive comments and suggestions on our manuscript entitled “Research on vertical bearing performance of inclined high-pressure rotary spray pile”. (ID: PONE-D-24-43810R1).
We have studied reviewer’s comments carefully and have made revisions which are marked in red in the paper. We have tried our best to revise our manuscript according to the comments and journal formatting guidelines. Attached please find the revised version, which we would like to submit for your kind consideration.
We would like to express our great appreciation to you and reviewers for comments on our paper. We are looking forward to hearing from you.
Thank you and best regards.
Yours sincerely,
Fei Gan
Corresponding author:
Name: Fei Gan
E-mail: [fgan@gzu.edu.cn](mailto:fgan@gzu.edu.cn)

List of Responses
Dear Editors and Reviewers:
Thank you for your letter and for the reviewers’ comments concerning our manuscript entitled “Research on vertical bearing performance of inclined high-pressure rotary spray pile”. (ID: PONE-D-24-43810R1). Those comments are all valuable and very helpful for revising and improving our paper, as well as the important guiding significance to our research. We have studied comments carefully and have made corrections which we hope meet with approval. Revised portions[1] are marked in red or blue in the paper. Original text in red italics. The main corrections in the paper and the responses to the reviewer’s comments are as follows:

**Responds to the reviewer’s comments:
Reviewer #1:**

1. Thanks for the responses. However, ‘Conclusion and Discussion’ are two different stories. You have discussed your results so; this part should be ‘conclusion’ or ‘concluding remarks’.

Response: Thank you very much. We have made the necessary modifications based on your feedback

（See line 345 on page 17 in the article）

2. Some inconsistencies within the reference list MUST be treated. 26 and 29 are the same and one of them MUST be removed or replaced. Double check the rest of references.

Response: Thank you very much for the reviewer's reminder. We have replaced Article 29. And checked the remaining references.

*[29]. Shahri A A, Moud F M (2021) Landslide susceptibility mapping using hybridized block modular intelligence model. BULLETIN OF ENGINEERING GEOLOGY AND THE ENVIRONMENT. 80(1) 267-284.* [*https://doi.org/10.1007/s10064-020-01922-8*](https://doi.org/10.1007/s10064-020-01922-8).

（See lines 458-460 on page 21 in the article）

3. Change the title and remove ‘search’.

Response: Thank you very much. We have revised the title based on your feedback.

*‘Vertical bearing performance of inclined high-pressure rotary spray pile’*

（See line 1 on page 1 in the article）

4. Please doublecheck the English to polish some minor typos.

Response: Thank you very much for your suggestion. We have made every effort to improve the manuscript. We have carefully checked the English and eliminated some minor spelling errors. We appreciate the reviewer's diligent work and hope the corrections will be approved.

**Reviewer # 2:**

1．Dear Auther(s)

Fig. 4 (According to the grain size distribution, the soil is sand, not clay).Please correct the curve or indicate the limits of both sand and clay size, citing the approved standard.

Response: Dear reviewer, thank you very much for your reminder. We have remeasured and recorded the particle size distribution data of the red clay, and plotted the grading curve of the red clay. We hope for your approval.

（See line 121 on page 6 in the article）
