# Supplementary material for: Vertical bearing performance of inclined high-pressure rotary spray pile
Source: PLoS One. 2025 Mar 27;20(3):e0319849. doi: 10.1371/journal.pone.0319849 (PMC11949337; doi:10.1371/journal.pone.0319849)
Supplement: Attachment — Submitted filename: Response_to_Reviewers_auresp_3.docx [file pone.0319849.s005.docx]

Dear editor:
On behalf of my co-authors, we thank you very much for giving us an opportunity to revise our manuscript, we appreciate editor and reviewers very much for their positive and constructive comments and suggestions on our manuscript entitled “Research on vertical bearing performance of inclined high-pressure rotary spray pile”. (ID: PONE-D-24-43810R2).
We have studied reviewer’s comments carefully and have made revisions which are marked in red in the paper. We have tried our best to revise our manuscript according to the comments and journal formatting guidelines. Attached please find the revised version, which we would like to submit for your kind consideration.
We would like to express our great appreciation to you and reviewers for comments on our paper. We are looking forward to hearing from you.
Thank you and best regards.
Yours sincerely,
Fei Gan
Corresponding author:
Name: Fei Gan
E-mail: [fgan@gzu.edu.cn](mailto:fgan@gzu.edu.cn)

List of Responses
Dear Editors and Reviewers:
Thank you for your letter and for the reviewers’ comments concerning our manuscript entitled “Research on vertical bearing performance of inclined high-pressure rotary spray pile”. (ID: PONE-D-24-43810R2). Those comments are all valuable and very helpful for revising and improving our paper, as well as the important guiding significance to our research. We have studied comments carefully and have made corrections which we hope meet with approval. Revised portions[1] are marked in red or blue in the paper. Original text in red italics. The main corrections in the paper and the responses to the reviewer’s comments are as follows:

**Responding to the editor's comments:**

Please review your reference list to ensure that it is complete and correct. If you have cited papers that have been retracted, please include the rationale for doing so in the manuscript text, or remove these references and replace them with relevant current references. Any changes to the reference list should be mentioned in the rebuttal letter that accompanies your revised manuscript. If you need to cite a retracted article, indicate the article’s retracted status in the References list and also include a citation and full reference for the retraction notice.

Response: Thanks for the editor's reminder. We have checked the reference list to ensure its completeness and accuracy.

**Responds to the reviewer’s comments:
Reviewer #1:**

1: Thanks for your cooperation and responding. Before publication, make a review to remove some minor typos.
Good luck

Response: Thank you very much for the patient response from the reviewer and also for your blessings. We have carefully checked the grammar in the article and removed some minor spelling errors. The modified parts have been indicated in red font in the article(Revised Manuscript with Track Changes). We hope the reviewer approves.

**Reviewer # 2:**

1. Dear Author(s)
   In figure 4, the soil is gravelly sand, not clay soil, according to Unified Soil Classification System (ASTM D2487-17e1)
   Gravel (>4.75 mm) = 100-64 = 36%
   Sand = 64%
   Clay = 0 %
   Please redraw the figure so that the passage through the No. 200 sieve is more than 50% according to the unified classification system, or set the grain sizes of gravel, sand and clay according to Chinese specifications

Response: Thank you very much for the reviewer's serious and patient response. We have redrawn the chart according to the reviewer's request, and we sincerely hope to receive your approval.

（See line 120 on page 6 in the article）
